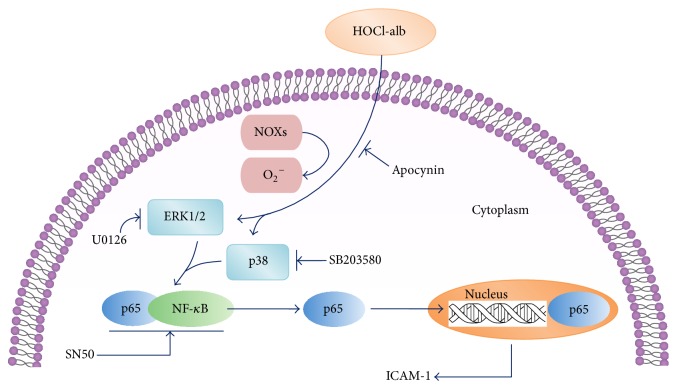# Hypochlorite-Modified Albumin Upregulates ICAM-1 Expression* via* a MAPK–NF-*κ*B Signaling Cascade: Protective Effects of Apocynin

**DOI:** 10.1155/2016/1852340

**Published:** 2016-01-10

**Authors:** Dong-dong Tang, Hong-xin Niu, Fen-fen Peng, Hai-bo Long, Zong-rui Liu, Hao Zhao, Yi-hua Chen

**Affiliations:** Division of Nephrology, Zhujiang Hospital, Southern Medical University, Guangzhou, Guangdong 510280, China

## Abstract

Hypochlorite-modified albumin (HOCl-alb) has been linked to endothelial dysfunction, which plays an important role in the development of hypertension, diabetes, and chronic kidney disease. However, whether HOCl-alb induces endothelial dysfunction* via* vascular inflammation and whether a signaling pathway is involved are unknown and have not been investigated. HOCl-alb was found to upregulate ICAM-1 expression in human umbilical vein endothelial cells (HUVECs) in a time- and dose-dependent manner. HOCl-alb time-dependently phosphorylated ERK1/2 and p38^MAPK^. HOCl-alb also activated NF-*κ*B. ICAM-1 expression was dose-dependently inhibited by U0126 (a specific inhibitor of MEK1/2, a signal upstream from ERK1/2), SB203580 (a specific inhibitor of p38^MAPK^), and SN50 (a specific inhibitor of NF-*κ*B). U0126 and SB203580 both counteracted the activation of NF-*κ*B, whereas the phosphorylation of ERK1/2 and p38^MAPK^ was not blocked by SN50. ERK1/2 phosphorylation was blocked by U0126 but not by SB203580, and p38^MAPK^ activity was reduced by SB203580 but not by U0126. Apocynin, a specific NADPH oxidase (NOX) inhibitor, inhibited ICAM-1 expression and the activity of ERK1/2, p38^MAPK^, and NF-*κ*B. These results indicate that HOCl-alb-induced ICAM-1 expression is caused by the activation of a redox-sensitive intracellular signal cascade involving ERK1/2 and p38^MAPK^, culminating in the activation of NF-*κ*B and involving NOXs among the upstream signals.

## 1. Introduction

Oxidative stress is caused by an imbalance between oxidants and antioxidants, when the former exceeds the latter, and manifests as the accumulation of large numbers of lipid and protein oxidation products, active aldehyde compounds, oxidative thiol, and carbonyl compounds. A newly recognized active compound, hypochlorite-modified albumin (HOCl-alb), is formed during the reaction between proteins and hypochlorite (HOCl) that originates from the myeloperoxidase of active neutrophils with the generation of oxidative stress and was isolated for the first time from uremic patients by Witko-Sarsat and his colleagues [1] in 1996. It was confirmed to be an aggregate of albumin likely resulting from disulfide bridges and/or dityrosine cross-linking. The spectral characteristic of this compound, with an obvious light absorption peak at a wavelength of 340 nm under acidic conditions, differs from that of natural albumin, which has a light absorption peak at 280 nm, indicating that the molecular structure and biochemical individuality of the natural albumin have been altered by the action of HOCl.* In vitro*, the interaction between HOCl and albumin can produce HOCl-modified albumin with the same biochemical characteristic as that extracted from uremic patients [1]. In addition to uremia, HOCl-alb has also been found in patients with hypertension [2], atherosclerosis [3], and diabetes [4, 5].

Endothelial cells cover the inner surfaces of blood vessels. They not only function as a selectively permeable barrier and in material exchange with the blood but also can directly perceive signals in the blood circulation and respond accordingly to maintain the normal functions of the blood vessels by synthesizing and secreting various bioactive substances. Under normal circumstances, blood vessels are in a relaxed state because the effects of vasodilators are stronger than those of vasoconstrictors. However, endothelial cell dysfunction causes an imbalance between vasodilators and vasoconstrictors, with a predominance of vasoconstriction, increased synthesis, and expression of a variety of proinflammatory factors by the damaged endothelial cells [6], and thrombosis, which is closely associated with a variety of diseases, including hypertension [7], atherosclerosis [6, 8], diabetes [9, 10], and chronic kidney disease [11].

Previous studies have shown that HOCl-alb causes endothelial dysfunction. In patients undergoing peritoneal dialysis, high levels of HOCl-alb correlate independently with increased oxidative stress markers and reduced vasodilatation function [12]. In rats with 5/6 nephrectomy, superoxide dismutase (SOD), a scavenger of superoxide radicals, ameliorates the increased levels of HOCl-alb, high blood pressure, and the reduced vasodilative response to acetylcholine [13].* In vitro*, HOCl-alb abolishes the endothelium-dependent relaxation of aortic rings [14]. These data show that HOCl-alb is associated with endothelial dysfunction and that the mechanisms may involve oxidative stress.

Nicotinamide adenine dinucleotide phosphate oxidases (NADPH oxidases, NOXs) are major reactive enzyme systems that produce reactive oxygen species and cause oxidative stress. Reactive oxygen species originating from NOXs can disturb the normal protective functions of the endothelium and are associated with the occurrence of vascular disease [15].

Previous data showed that HOCl-alb promoted proinflammatory mediator monocyte chemoattractant protein-1 (MCP-1) upregulation in HEK 293 cell lines [16] and in podocytes [17] and promoted MCP-1 expression* via* NOX in diabetic animals [18]. Whether HOCl-alb induces the expression of vascular inflammatory factors and the detailed molecular mechanism involved are still unclear. In this study, we investigated the signaling cascade triggered by HOCl-alb that upregulates ICAM-1 expression in human umbilical vein endothelial cells (HUVECs) and whether an inhibitor of NOXs, apocynin, inhibits the biopathological effects of HOCl-alb.

## 2. Materials and Methods

### 2.1. Reagents

RPMI 1640 was obtained from HyClone, Logan, Utah, USA; human group AB serum from the Biological Treatment Center, Nanfang Hospital, Guangzhou, Guangdong, China; fatty acid-free bovine serum albumin (Alb), propidium iodide (PI), SOD, diphenyleneiodonium (DPI), and apocynin from Sigma-Aldrich, St. Louis, MO, USA; SB203580 (a specific p38^MAPK^ inhibitor) and NF-*κ*B oligonucleotides from Promega, Madison, WI, USA; SN50 (a specific NF-*κ*B inhibitor) from Alexis Biochemicals, San Diego, CA, USA; mouse anti-human Actin and ICAM-1 antibody from Santa Cruz Biotechnology, Inc., Santa Cruz, CA, USA; rabbit anti-human NF-*κ*B/p65 antibody from BioVision, Mountain View, CA, USA; rabbit anti-human phospho-p38, rabbit anti-human p38, rabbit anti-human phospho-ERK1/2, rabbit anti-human ERK1/2, rabbit anti-human phospho-SAPK/JNK, rabbit anti-human SAPK/JNK, HRP-conjugated rabbit anti-mouse IgG, HRP-conjugated goat anti-rabbit IgG antibodies, and U0126 (a specific MEK1/2 inhibitor) from Cell Signaling Technology, Beverly, MA, USA; fluorescein isothiocyanate- (FITC-) conjugated rabbit anti-mouse IgG and FITC-conjugated swine anti-rabbit IgG from DakoCytomation, Glostrup, Denmark; PRC primers from Sangon, Shanghai, China; Trizol Reagent from Invitrogen, USA; an RT-PCR kit from TaKaRa, Japan; a Reactive Oxygen Species (ROS) Assay Kit from Beyotime, China; and Gel Mobility Shift Assay Kits from Roche Applied Science, Mannheim, Germany.

### 2.2. Isolation and Culture of HUVECs

HUVECs were isolated and cultured as described previously [19] with some modification. Briefly, healthy neonatal human umbilical cords, 20–35 cm in length, were stored at 4°C for no more than 12 h, and then 0.5 cm was removed from each end. The cords were then lavaged three times with cold phosphate-buffered saline (PBS) until the effluent liquid was clear. The umbilical cords were filled with about 10–15 mL of digestive juices containing 0.25% trypsin and 0.02% EDTA. After incubation at 37°C for 8–10 min, the digestive juices were collected into a 50 mL centrifuge tube containing RPMI 1640 with 10% fetal blood serum and then centrifuged at 300 ×g for 10 min. The supernatant was removed, and RPMI 1640 containing 20% human group AB serum was added to the centrifuge tube and mixed with the cell sediment. The cells were adjusted to a concentration of 1.5–2.0 × 10^4^/cm^2^ and plated in a culture bottle at 37°C under 5% CO_2_. The cells were grown in RPMI 1640 containing 20% human group AB serum, 80 U/mL penicillin, and 80 *μ*g/mL streptomycin. First-or second-generation cells were used for the experiments.

### 2.3. Preparation of HOCl-alb

HOCl-alb was prepared* in vitro* as described previously [1, 20] and by ourselves [17, 18]. Equivalent volumes of 20 g/L fatty acid free albumin and 40 mM HOCl were mixed for 30 min at room temperature, and HOCl-alb was prepared in molar ratios of 1 : 140. A mixture of equivalent volumes of 20 g/L Alb and PBS was used as the control. The prepared HOCl-alb and unmodified Alb without modification were dialyzed overnight against PBS to remove any free HOCl, sterilized by filtration through a 0.22 *μ*m microporous membrane, and stored at 4°C. The HOCl-alb content was measured as the absorbance at 340 nm under acid conditions, based on a chloramine-T standard. The HOCl-alb contents in the HOCl-alb and unmodified Alb preparations were 32.68 *μ*M and 1.56 *μ*M, respectively. After the protein concentration was corrected, they were 4.58 *μ*mol/g and 0.15 *μ*mol/g, respectively.

The preparation was conducted in the absence of free amino acids/carbohydrates/lipids to exclude the formation of AGE-like structures. A limulus test showed that the content of endotoxin in both preparations was <0.25 EU/mL.

### 2.4. Detection of ICAM-1

#### 2.4.1. Determination of ICAM-1 mRNA with Reverse Transcription-PCR (RT-PCR)

Confluent HUVECs were stimulated with different concentrations of HOCl-alb or 200 mg/L unmodified Alb for 6 h. In another set of cells, 200 mg/L HOCl-alb was added and the cells were collected at different time points. Trizol Reagent (Invitrogen) was used to extract the total RNAs. RT-PCR was performed according to the manufacturer's instructions. *β*-Actin was used as the internal reference. The primer sequences used were ICAM-1 sense strand 5′-TATGGCAACGACTCCTTCT-3′, antisense strand 5′-CCAAGGTGACGCTGAATG-3′, amplifying a fragment of 238 bp; *β*-Actin sense strand 5′-GACTACCTCATGAAGATCCT-3′, and antisense strand 5′-CCACATCTGCTGGAAGGTGG-3′, amplifying a fragment of 510 bp. The amplified PCR products were evaluated with 2% agarose gel electrophoresis with ethidium bromide staining. The Quantity One gel image analysis system (Bio-Rad, Hercules, CA, USA) was used to analyze the results.

#### 2.4.2. Determination of ICAM-1 Protein Expression by Western Blot

HUVECs were stimulated with different concentrations of HOCl-alb or 200 mg/L unmodified Alb for 12 h, and 200 mg/L HOCl-alb was added to other sets of cells and incubated for different periods of time.

To understand the influence of NOXs, mitogen-activated protein kinases (MAPKs), and NF-*κ*B in the expression of ICAM-1 induced by HOCl-alb, HUVECs were preincubated with different concentrations of apocynin [21], DPI [22], SOD [23], U0126, SB203580 [24], or SN50 [25] for 1 h, followed by the addition of 200 mg/L HOCl-alb for 12 h. A methyl thiazolyl tetrazolium (MTT) assay showed that all inhibitors employed had no effects on cell survival (data not shown). The cells were collected and the total protein was extracted. Western blotting was used to determine the ICAM-1 protein content. The Quantity One gel image analysis system was used to analyze the results.

#### 2.4.3. Localization of ICAM-1 Protein in HUVECs with Immunofluorescence

HUVECs were incubated with 200 mg/L HOCl-alb or unmodified Alb for 12 h. After the cells were washed twice with PBS, they were fixed with methanol : acetone (1 : 1) at −20°C for 10 min. The cells were washed three times with PBS, blocked in 1% bovine serum albumin at room temperature for 30 min, incubated with mouse anti-human ICAM-1 antibody (diluted 1 : 100), and vibrated in a wet box overnight at 4°C. After the cells were washed, they were incubated with FITC-conjugated rabbit anti-mouse IgG (diluted 1 : 64) in the dark at 37°C for 45 min. After the cells were washed, they were incubated with 10 *μ*g/mL PI in the dark at room temperature for 10 min, washed three times with PBS, sealed with buffered glycerol, stored in the dark at 4°C, and observed under a confocal microscope.

### 2.5. Detection of MAPK Phosphorylation

HUVECs were treated with 200 mg/L HOCl-alb for 0, 5, 10, 15, 30, and 60 min and collected for protein extraction.

Another group of cells was pretreated with 500 *μ*M apocynin, 10 *μ*M DPI, 200 U/mL SOD, 10 *μ*M U0126, 10 *μ*M SB203580, or 50 mg/L SN50 for 1 h and then incubated with 200 mg/L HOCl-alb for 15 min. The cells were collected and their total protein was extracted.

Western blotting was used to detect phospho-ERK1/2, ERK1/2, phospho-p38^MAPK^, and p38^MAPK^. The results were analyzed with the Quantity One gel image analysis system.

### 2.6. Detection of NF-*κ*B Activation

#### 2.6.1. Western Blot

HUVECs were incubated with 200 mg/L HOCl-alb and collected after 0, 15, 30, 60, and 120 min. The nuclear and cytoplasmic proteins were extracted with methods reported in the literature [26].

Another group of cells was pretreated with apocynin, U0126, SB203580, or SN50 for 1 h before stimulation with 200 mg/L HOCl-alb for 30 min. The cells were then collected for nuclear and cytoplasmic protein extraction.

The amounts of nuclear p65 and cytoplasmic p65 proteins were determined with western blotting.

#### 2.6.2. Electrophoretic Mobility Shift Assay (EMSA)

HUVECs were treated and their nuclear and cytoplasmic proteins were extracted as described above. The nuclear translocation of NF-*κ*B and its subsequent binding to DNA were measured with EMSA. The oligonucleotide probe for NF-*κ*B (sequence: 5′-AGTTGAGGGGACTTTCCCAGGC-3′ and 3′-TCAACTCCCCTGAAAGGGTCCG-5′) was labeled with digoxigenin. In the binding reaction system (4 *μ*L of binding buffer, 1 *μ*g of poly-d(I-C), 0.1 *μ*g poly-l-lysine, 10 *μ*g of sample nucleoprotein, and 3.5 pmol of oligonucleotide probe), the NF-*κ*B in the nucleoprotein sample was combined with the labeled oligonucleotide probe. A competition assay was performed with a 125x excess of unlabeled NF-*κ*B oligonucleotide probe added into the reaction system for 1 h to detect the specificity of the reaction. After the reaction, 5 *μ*L of loading buffer was added to the sample, resolved with 6% native polyacrylamide gel electrophoresis, and electrotransferred to a positively charged nylon membrane. The membrane was baked at 120°C for 20 min. After the membrane was rinsed and blocked, an alkaline-phosphatase-labeled anti-digoxigenin antibody and CSPD were added sequentially for development. The Quantity One gel image analysis system was used to observe and analyze the results.

#### 2.6.3. Immunofluorescence

HUVECs were stimulated with 200 mg/L HOCl-alb for 30 min. After the cells were fixed, rinsed, and blocked, they were incubated overnight at 4°C with rabbit anti-human NF-*κ*B/p65 antibody (10 *μ*g/mL). After washing, the cells were incubated in FITC-labeled swine anti-rabbit IgG (1 : 20) at 37°C in the dark for 45 min and then incubated with PI (10 *μ*g/mL) at room temperature in the dark for 10 min. The cells were observed and photographed with confocal microscopy (Leica TCS SP2 AOBS, Leica Microsystems, Cambridge, UK).

### 2.7. Detection of ROS Generation

#### 2.7.1. Determination of Total Intracellular ROS with DCFDA Fluorescence Intensity

Total intracellular ROS were measured using a ROS Assay Kit according to the manufacturer's manual. Briefly, HUVECs were preincubated for 30 min with 1 nM 2,7-dichlorofluorescin diacetate (DCFDA) and then incubated with 200 mg/L HOCl-alb for 15 min in the presence or absence of apocynin, DPI, or SOD at the indicated concentrations. Total intracellular ROS were determined by measuring the fluorescence intensity of DCFDA on a flow cytometry (BD FACSCalibur system, Franklin Lakes, NJ, USA).

#### 2.7.2. Determination of Superoxide in Homogenates by Lucigenin-Enhanced Chemiluminescence Assay

The lucigenin-enhanced chemiluminescence assay was used to measure superoxide in homogenates of HUVECs as described previously [27]. Briefly, the cells were preincubated with apocynin, DPI, or SOD at the indicated concentrations for 1 h and treated with 200 mg/L HOCl-alb for 15 min. Cell homogenates (100 *μ*g/well) were added into a 96-well microplate and exposed to dark-adapted lucigenin (5 *μ*M). Light emission was recorded every min for 30 min (VICTOR V Wallac 1420, PerkinElmer, Turku, Finland). Data were expressed as light units relative to the control group.

### 2.8. Statistical Analysis

All experiments were performed three or more times. Continuous variables were expressed as means ± SD. The overall differences and those among groups were compared with one-way analysis of variance (ANOVA), followed by the Student-Newman-Keuls test and least significant difference (LSD), between two groups. Two-tailed *p* values <0.05 were considered statistically significant. All statistical analyses were performed with the SPSS 13.0 software (SPSS, Chicago, IL, USA).

## 3. Results

### 3.1. HOCl-alb Upregulated ICAM-1 Expression in HUVECs

#### 3.1.1. Time and Dose Effects of HOCl-alb on ICAM-1 Protein Expression in HUVECs

**Figure 1:**
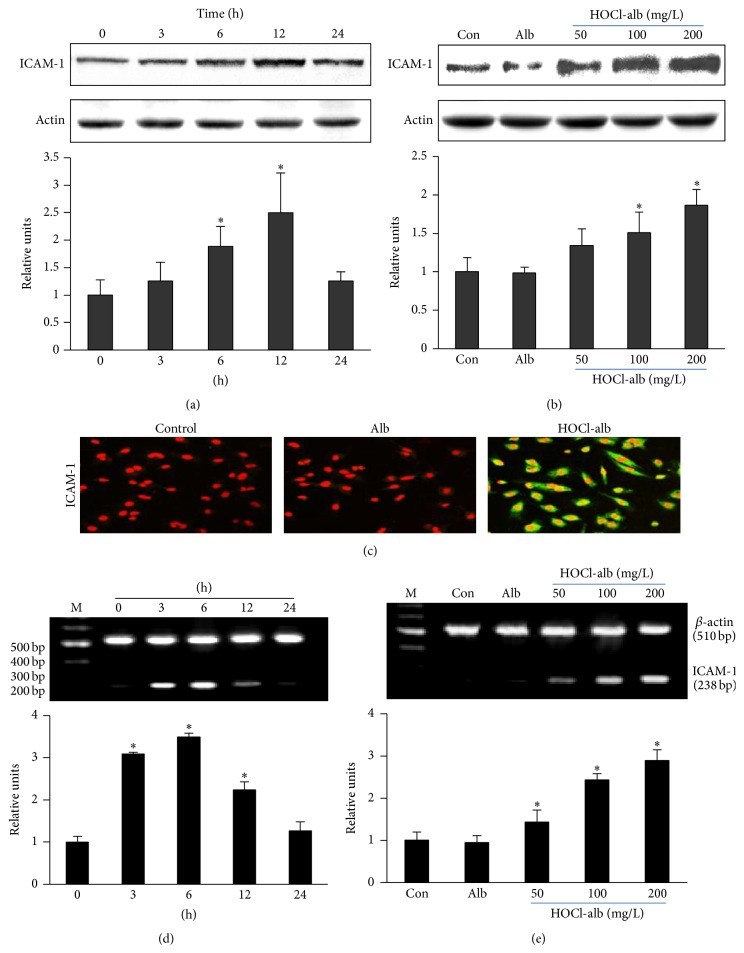
Effects of HOCl-alb on ICAM-1 expression in HUVECs. HUVECs were stimulated with the indicated concentrations of HOCl-alb or 200 mg/L unmodified Alb for the indicated times, and then their ICAM-1 protein and mRNA expression were analyzed. HOCl-alb treatment increased ICAM-1 expression at both the protein (a, b, c) and mRNA levels (d, e) in a dose- and time-dependent manner. Data are expressed as the means ± SD of three independent experiments. ANOVA, *p* < 0.01 in (a), (b), (d), (e); ^*∗*^ *p* < 0.05 versus the control (b, e) or 0 h (a, d).

HOCl-alb upregulated ICAM-1 expression in HUVECs in a time-dependent manner. A small amount of ICAM-1 was expressed in HUVECs under basal conditions. Compared with baseline, the expression of ICAM-1 increased slightly at 3 h (1.26 ± 0.34-fold, *p* = 0.470) after stimulation with 200 mg/L HOCl-alb, had changed significantly at 6 h (1.88 ± 0.68-fold, *p* = 0.027), and peaked at 12 h (2.50 ± 0.72-fold, *p* = 0.001). ICAM-1 expression then decreased gradually but was still slightly higher (1.26 ± 0.17-fold, *p* = 0.470) than the basal level (Figure 1(a)).

The expression of ICAM-1 protein in HUVECs also increased when the concentration of HOCl-alb increased. When HUVECs were incubated with 50, 100, or 200 mg/L HOCl-alb for 12 h, cellular ICAM-1 protein production increased 1.34 ± 0.22-fold (*p* = 0.060), 1.51 ± 0.26-fold (*p* = 0.010), and 1.87 ± 0.20-fold (*p* < 0.001), respectively, relative to that of the control group. Unmodified Alb had no obvious effect on ICAM-1 protein expression (*p* = 0.935) (Figure 1(b)).

#### 3.1.2. Cellular Localization of ICAM-1 Protein Induced by HOCl-alb in HUVECs

Immunofluorescent staining showed that ICAM-1 protein was expressed both on the cell membrane and in the cytoplasm of HUVECs. ICAM-1 expression increased and fluorescence intensity was clearly enhanced after the cells were incubated with 200 mg/L HOCl-alb for 12 h. Unmodified Alb had no significant effect on ICAM-1 expression (Figure 1(c)).

#### 3.1.3. Effect of HOCl-alb on ICAM-1 mRNA Expression in HUVECs

Stimulation of cells with 200 mg/L HOCl-alb for 3 h significantly increased the expression of ICAM-1 mRNA to 3.08 ± 0.04-fold the basal level (*p* < 0.001) and peaked at 3.48 ± 0.10-fold after stimulation for 6 h (*p* < 0.001). ICAM-1 mRNA expression then gradually declined and approached the baseline level at 24 h (*p* = 0.060) (Figure 1(d)).

The stimulatory effect of HOCl-alb on ICAM-1 mRNA increased as the concentration of HOCl-alb increased. The incubation of HUVECs with 50, 100, or 200 mg/L of HOCl-alb for 6 h increased the cellular ICAM-1 mRNA expression 1.43 ± 0.29-fold (*p* = 0.036), 2.43 ± 0.15-fold (*p* < 0.001), and 2.89 ± 0.25-fold (*p* < 0.001), respectively, relative to the control. Unmodified Alb had no effect on ICAM-1 mRNA expression (*p* = 0.752) (Figure 1(e)).

### 3.2. HOCl-alb Activated ERK1/2 and p38^MAPK^ in HUVECs

**Figure 2:**
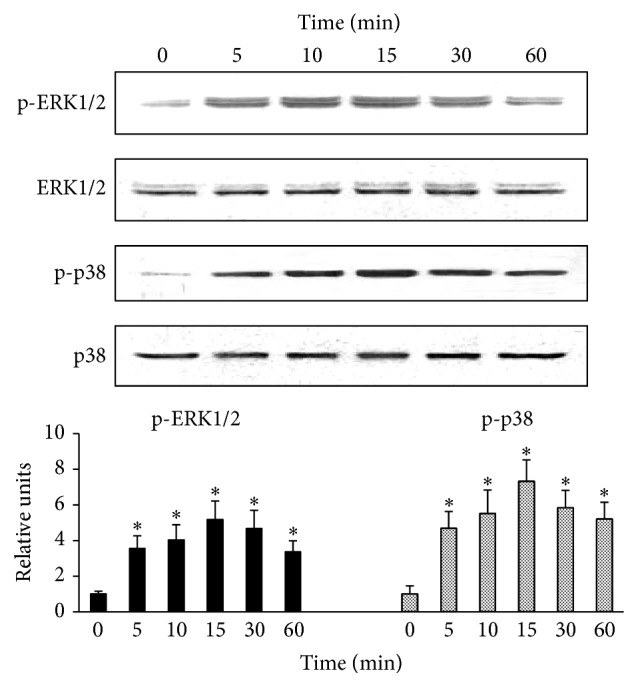
HOCl-alb induced the phosphorylation of ERK1/2 and p38^MAPK^ in HUVECs. HUVECs were stimulated with 200 mg/L HOCl-alb for the indicated times and the phosphorylation of ERK1/2 and p38^MAPK^ was determined with western blotting using anti-phospho-ERK1/2, anti-ERK1/2, anti-phospho-p38^MAPK^, and anti-p38^MAPK^ primary antibodies. Data are expressed as the means ± SD of three independent experiments. ANOVA, *p* < 0.01; ^*∗*^ *p* < 0.05 versus 0 min.

HOCl-alb caused strong time-dependent phosphorylation of ERK1/2 and p38^MAPK^. After stimulation with 200 mg/L HOCl-alb for 5 min, ERK1/2 was rapidly activated, with its phosphorylation increasing 3.56 ± 0.7-fold (*p* = 0.002) relative to the baseline level. ERK1/2 phosphorylation peaked at 15 min, at a level of 5.17 ± 1.04-fold higher than the baseline level (*p* < 0.001). It then decreased gradually but was still elevated at 60 min, at 3.37 ± 0.61-fold the baseline level (*p* = 0.003). Total ERK1/2 protein expression was not altered. The time course of p38^MAPK^ activation in the HOCl-alb-treated cells was similar to that of ERK1/2. When cells were stimulated with HOCl-alb for 5, 10, 15, 30, or 60 min, p38^MAPK^ phosphorylation was 4.68 ± 0.94-fold (*p* = 0.001), 5.51 ± 1.32-fold (*p* < 0.001), 7.32 ± 1.2-fold (*p* < 0.001), 5.83 ± 0.98-fold (*p* < 0.001), and 5.2 ± 0.94-fold higher, respectively, than the baseline level (*p* < 0.001). The total expression of p38^MAPK^ protein was not altered (Figure 2). HOCl-alb (200 mg/L) had no effect on JNK/SAPK phosphorylation (data not shown).

### 3.3. HOCl-alb Activated NF-*κ*B in HUVECs

**Figure 3:**
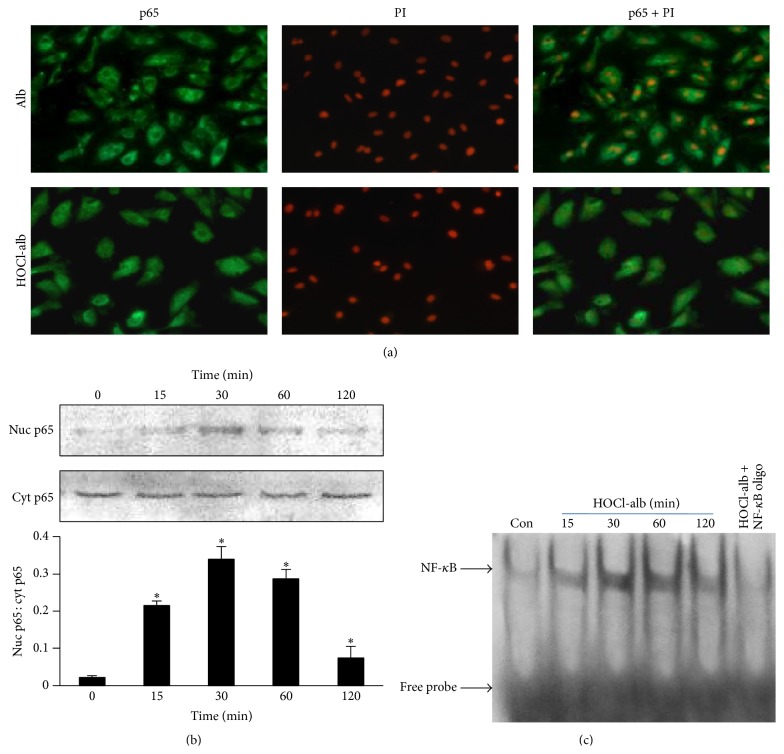
HOCl-alb induced the activation of NF-*κ*B in HUVECs. (a) HUVECs were stimulated with 200 mg/L HOCl-alb for 30 min. Translocation of NF-*κ*B/p65 was detected with immunofluorescence. Cells were incubated with rabbit anti-human NF-*κ*B/p65 antibody (10 *μ*g/mL) overnight at 4°C, FITC (green) labeled swine anti-rabbit IgG (1 : 20) at 37°C for 45 min, and 10 *μ*g/mL PI (red) at room temperature in the dark for 10 min and then observed under a confocal microscope at 10 × 40 times magnification. (b) HUVECs were stimulated with 200 mg/L HOCl-alb for the indicated times. NF-*κ*B/p65 was detected in the nuclear extracts (nuc p65) and cytoplasmic extracts (cyt p65) with western blotting. (c) HOCl-alb-induced NF-*κ*B activation in the nuclear extracts was detected with EMSA. Data are expressed as the means ± SD of three independent experiments. ANOVA, *p* < 0.01 in (b); ^*∗*^ *p* < 0.05 versus 0 min.

NF-*κ*B, an oxidant-sensitive transcription factor, plays an important role in regulating the inflammatory response. Immunofluorescent staining and confocal microscopy showed that 200 mg/L HOCl-alb induced translocation of the NF-*κ*B p65 subunit (green fluorescence) from the cytoplasm to the nucleus (Figure 3(a)).

We monitored the time course of NF-*κ*B activation by 200 mg/L HOCl-alb with western blotting. After stimulation with HOCl-alb, the NF-*κ*B p65 subunit translocated from the cytoplasm to the nucleus in a time-dependent manner. The exposure of cells to 200 mg/L HOCl-alb for 15 min induced marked nuclear translocation of the NF-*κ*B p65 subunit, with a 9.69-fold increase in the amount of protein in the nucleus relative to the basal state (*p* < 0.001). The translocation of NF-*κ*B peaked after exposure to 200 mg/L HOCl-Alb for 30–60 min, which was 15.30-fold (*p* < 0.001) and 12.95-fold (*p* < 0.001) higher than the basal level, respectively. The translocation of NF-*κ*B then declined but was still higher than the baseline value. The amount of NF-*κ*B p65 translocated into the nucleus at 120 min was 3.36-fold compared to that at 0 min (*p* = 0.02) (Figure 3(b)). EMSA confirmed the activation of NF-*κ*B by HOCl-alb. Accompanying the translocation of NF-*κ*B, the binding of NF-*κ*B to DNA began at 15 min and peaked at 30–60 min after stimulation with HOCl-alb. The shifted band disappeared after the addition of excess unlabeled oligonucleotide probe for NF-*κ*B into the reaction system, but the addition of a control oligonucleotide had no effect on the translocation of NF-*κ*B, confirming the specificity of the reaction (Figure 3(c)).

### 3.4. Effects of ERK1/2, p38^MAPK^, and NF-*κ*B on HOCl-alb-Induced ICAM-1 Upregulation

**Figure 4:**
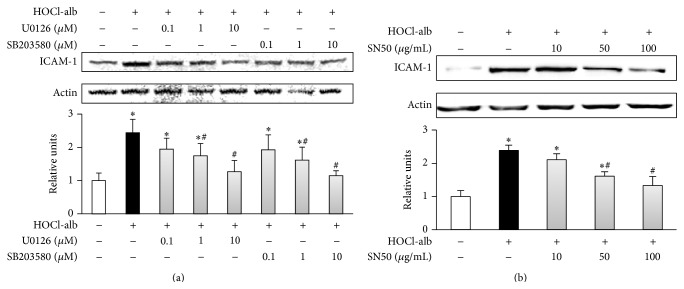
Effects of MAPKs and NF-*κ*B on HOCl-alb-induced ICAM-1 expression. HUVECs were preincubated with the indicated concentrations of U0126, SB203580 (a), or SN50 (b) for 1 h before stimulation with 200 mg/L HOCl-alb for 12 h. ICAM-1 protein expression was analyzed with western blotting. Data are expressed as the means ± SD of three independent experiments. ANOVA, *p* < 0.01 in (a) and (b); ^*∗*^ *p* < 0.05 versus control, ^#^ *p* < 0.05 versus the HOCl-alb group.

To further investigate the effects of ERK1/2, p38^MAPK^, and NF-*κ*B on the HOCl-alb-induced upregulation of ICAM-1, U0126, a specific inhibitor of MEK1/2 (a signal molecule upstream from ERK1/2), SB203580, a specific inhibitor of p38^MAPK^, and SN50, a specific inhibitor of NF-*κ*B, were added to HUVECs before stimulation with HOCl-alb. U0126, SB203580, and SN50 all inhibited the HOCl-alb-induced expression of ICAM-1, in a manner related to the concentration of the inhibitor. U0126 (0.1 *μ*M) had no effect on HOCl-alb-upregulated ICAM-1 expression (*p* = 0.098, compared with the HOCl-alb group), whereas 1 *μ*M and 10 *μ*M U0126 significantly inhibited ICAM-1 expression, at rates of 28.57% (*p* = 0.026) and 48.16% (*p* < 0.001), respectively. The effect of SB203580 on ICAM-1 expression was similar to that of U0126; 0.1 *μ*M SB203580 had no effect on the upregulation of ICAM-1 expression (*p* = 0.086, compared with the HOCl-alb group), whereas 1 *μ*M and 10 *μ*M SB203580 clearly inhibited HOCl-alb-induced ICAM-1 expression by 33.88% (*p* = 0.010) and 53.06% (*p* < 0.001), respectively (Figure 4(a)). SN50 (10 *μ*g/mL) had no effect on the induction of ICAM-1 expression by HOCl-alb (*p* = 0.095, compared with the HOCl-alb group), whereas 50 *μ*g/mL and 100 *μ*g/mL SN50 clearly inhibited the HOCl-alb-induced ICAM-1 expression by 32.65% (*p* = 0.001) and 44.44% (*p* < 0.001), respectively (Figure 4(b)). This suggests that ERK1/2, p38^MAPK^, and NF-*κ*B are involved in the upregulation of ICAM-1 expression by HOCl-alb in HUVECs.

### 3.5. Relationships between ERK1/2, p38^MAPK^, and NF-*κ*B in the Signal Transduction Underlying HOCl-alb-Induced ICAM-1 Expression in HUVECs

**Figure 5:**
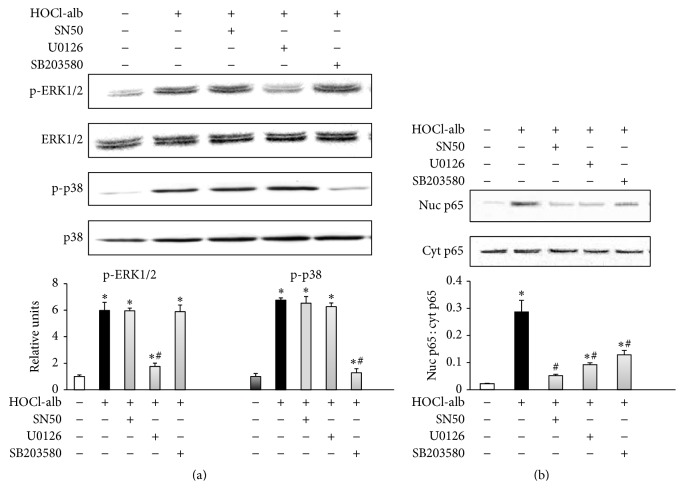
Relationships between ERK1/2, p38^MAPK^, and NF-*κ*B. HUVECs were preincubated with SN50, U0126, or SB203580 for 1 h before stimulation with 200 mg/L HOCl-alb. The activation of ERK1/2 and p38^MAPK^ (a) and the proportions of NF-*κ*B/p65 in the nucleus and cytoplasm (b) were determined with western blotting. Data are expressed as the means ± SD of three independent experiments. ANOVA, *p* < 0.01 in (a) and (b); ^*∗*^ *p* < 0.05 versus control; ^#^ *p* < 0.05 versus the HOCl-alb group.

The experiments described above confirmed that ERK1/2, p38^MAPK^, and NF-*κ*B all play a role in the cellular signal transduction underlying the HOCl-alb-induced expression of ICAM-1. To clarify the association between ERK1/2, p38, and NF-*κ*B, specific inhibitors of these signal molecules were added to the cells before they were treated with HOCl-alb, and the contents of these signaling molecules in the cells were determined. We found that 10 *μ*M U0126 (a specific MEK1/2 inhibitor) and SB203580 (a specific p38 inhibitor) both counteracted the HOCl-alb-induced activation of NF-*κ*B (*p* < 0.001, compared with the HOCl-alb group) (Figure 5(b)), whereas the HOCl-alb-induced phosphorylation of ERK1/2 (*p* = 0.941, compared with the HOCl-alb group) and p38^MAPK^ (*p* = 0.379, compared with the HOCl-alb group) was not blocked by SN50, an NF-*κ*B inhibitor (Figure 5(a)). This indicates that the activation of NF-*κ*B involves ERK1/2 and p38^MAPK^, but the activation of ERK1/2 and p38^MAPK^ does not require NF-*κ*B.

To clarify the mutual relationship between ERK1/2 and p38^MAPK^, cells were preincubated with U0126 or SB203580 before they were treated with HOCl-alb, and the phosphorylation of ERK1/2 or p38^MAPK^ was then determined. HOCl-alb-induced ERK1/2 phosphorylation was blocked by U0126 (*p* < 0.001, compared with the HOCl-alb group) but not by SB203580 (*p* = 0.775, compared with the HOCl-alb group), whereas p38^MAPK^ activity was reduced by SB203580 (*p* < 0.001, compared with the HOCl-alb group) but not by U0126 (*p* = 0.077, compared with the HOCl-alb group), indicating that p38^MAPK^ is not required for ERK1/2 activation, and* vice versa* (Figure 5(a)).

These results showed that the ICAM-1 expression induced by HOCl-alb involved the MAPK–NF-*κ*B signaling cascade.

### 3.6. Effects of NOXs and Apocynin on the Signal Transduction Underlying HOCl-alb-Induced ICAM-1 Expression in HUVECs

**Figure 6:**
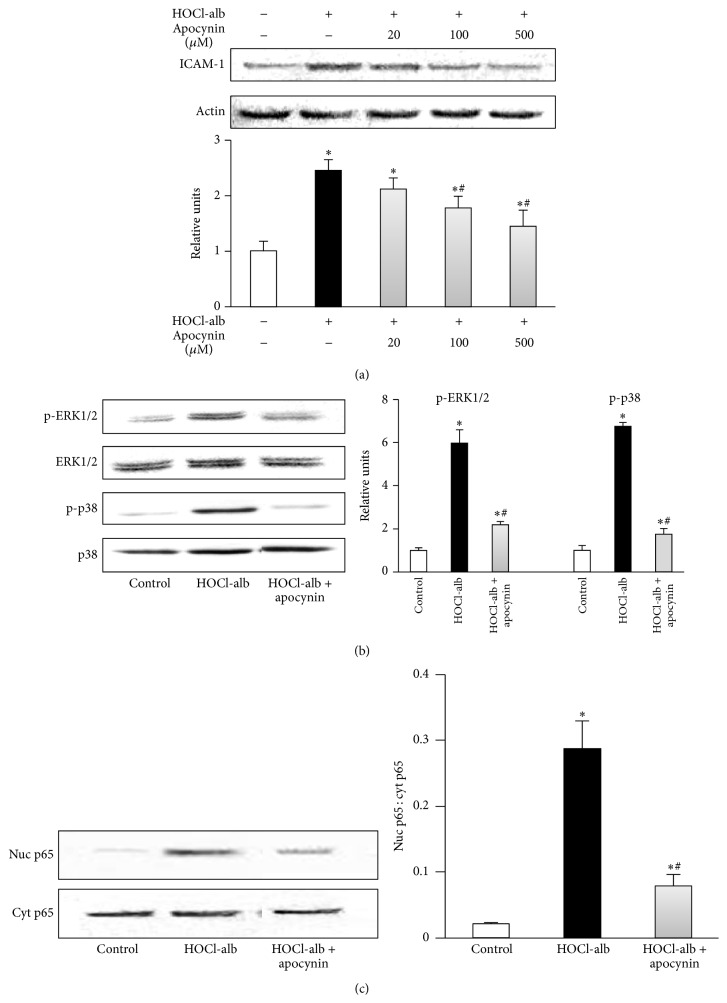
Effects of apocynin on signal transduction underlying HOCl-alb-induced ICAM-1 expression in HUVECs. (a) HUVECs were preincubated with the indicated concentrations of apocynin for 1 h and stimulated with 200 mg/L HOCl-alb for 12 h. ICAM-1 protein expression was analyzed with western blotting. (b) HUVECs were preincubated with 500 *μ*M apocynin for 1 h and then stimulated with 200 mg/L HOCl-alb for 15 min. The activation of ERK1/2 and p38^MAPK^ were determined with western blotting. (c) HUVECs were incubated with 500 *μ*M apocynin for 1 h and stimulated with 200 mg/L HOCl-alb for 30 min. Western blotting was used to detect the nuclear p65 and cytoplasmic p65. Data are expressed as the means ± SD of three independent experiments. ANOVA, *p* < 0.001 in (a), (b), and (c). ^*∗*^ *p* < 0.05 versus control; ^#^ *p* < 0.05 versus the HOCl-alb group.

Cells were preincubated with apocynin, a specific NOX inhibitor [18, 20], before they were treated with HOCl-alb. Our results showed that 20~500 *μ*M apocynin inhibited upregulation of ICAM-1 expression induced by HOCl-alb in a dose-dependent manner. 20 *μ*M apocynin had no significant effect on ICAM-1 expression induced by HOCl-alb (*p* = 0.097, compared with the HOCl-alb group). 100 and 500 *μ*M apocynin inhibited the upregulation of ICAM-1 expression induced by HOCl-alb by 46.21% (*p* = 0.004) and 68.97% (*p* < 0.001, compared with the HOCl-alb group), respectively (Figure 6(a)).

Cells were preincubated with 500 *μ*M apocynin for 1 h, and then 200 mg/L HOCl-alb was added. The phosphorylation activity of both ERK1/2 and p38^MAPK^ induced by HOCl-alb was inhibited by apocynin (*p* < 0.001, compared with the HOCl-alb group) (Figure 6(b)). Apocynin also inhibited NF-*κ*B activity (*p* < 0.001, compared with the HOCl-alb group) (Figure 6(c)).

**Figure 7:**
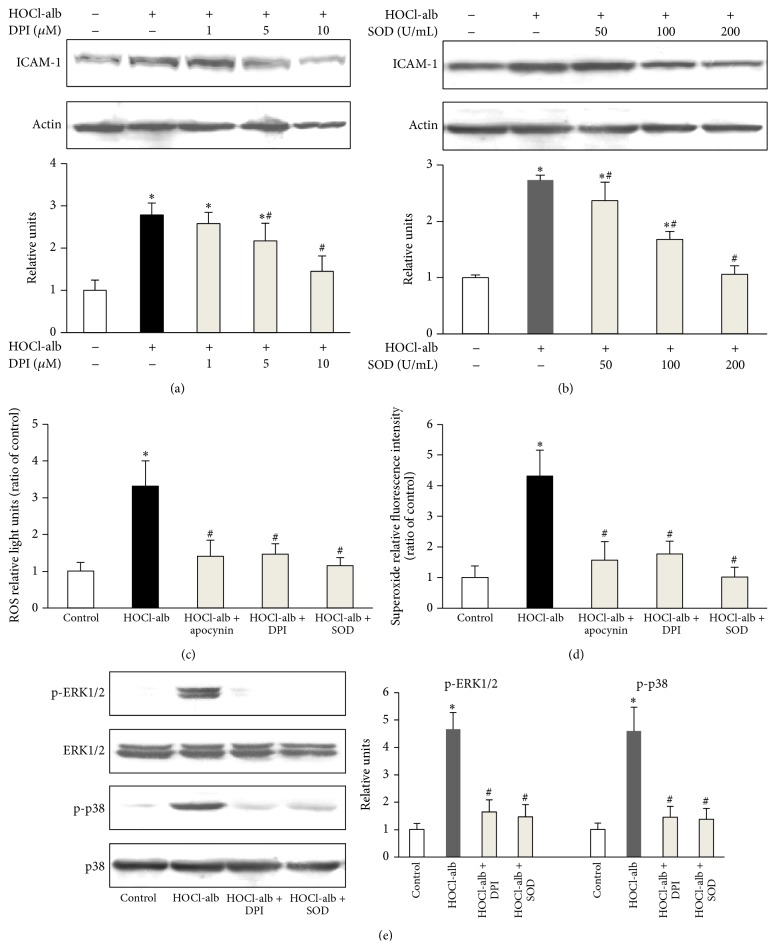
Effects of DPI and SOD on signal transduction underlying HOCl-alb-induced ICAM-1 expression, ROS generation, and phosphorylation of ERK1/2 and p38^MAPK^ in HUVECs. ((a) and (b)) HUVECs were preincubated with the indicated concentrations of DPI (a) or SOD (b) for 1 h and stimulated with 200 mg/L HOCl-alb for 12 h. ICAM-1 protein expression was analyzed with western blotting. ((c), (d), and (e)) HUVECs were preincubated with apocynin, DPI, or SOD for 1 h and then stimulated with 200 mg/L HOCl-alb. Total intracellular ROS was measured with DCFDA fluorescence intensity (c), superoxide in cell homogenates was detected by Lucigenin-enhanced chemiluminescence assay (d), and the activation of ERK1/2 and p38^MAPK^ were determined with western blotting (e). Data are expressed as the means ± SD of three independent experiments. ANOVA, *p* < 0.001 in (a), (b), (c), (d) and (e). ^*∗*^ *p* < 0.05 versus control; ^#^ *p* < 0.05 versus the HOCl-alb group.

1, 5, and 10 *μ*M DPI, an inhibitor of NOXs, inhibited HOCl-alb-induced ICAM-1 expression in a dose-dependent manner (Figure 7(a)). SOD, a scavenger of superoxide, also dose-dependently inhibited the upregulation of ICAM-1 expression induced by HOCl-alb (Figure 7(b)). Total intracellular ROS production, as determined by DCFDA fluorescence intensity, was significantly increased following exposure of HUVECs to 200 mg/L HOCl-alb (Figure 7(c)). To further confirm the intracellular source of ROS, lucigenin-enhanced chemiluminescence was used to determine superoxide in cell homogenates (Figure 7(d)). Both total intracellular ROS and superoxide induced by HOCl-alb were significantly prevented by apocynin, DPI, and SOD (Figures 7(c) and 7(d)). The phosphorylation activity of both ERK1/2 and p38^MAPK^ induced by HOCl-alb was inhibited by DPI and SOD (Figure 7(e)). These results suggested that NOXs were involved in the HOCl-alb-induced upregulation of ICAM-1 expression in HUVECs and that the activation of ERK1/2, p38^MAPK^, and NF-*κ*B required NOXs.

## 4. Discussion

This study demonstrated that HOCl-alb upregulated ICAM-1 in human vascular endothelial cells and highlighted the mechanisms involved. Diverse protein molecules including low density lipoproteins [28] have been the target of myeloperoxidase-derived HOCl to form modified products different from HOCl-alb in molecular structure, biochemical properties, and biological effects [1]. Albumin is the major plasma protein target of HOCl [16, 29]. We focused on HOCl modified albumin in the present study. In preliminary experiments, we prepared HOCl-alb with different degrees of modification according to the molar ratios of 1 : 70, 1 : 140, and 1 : 280 (albumin to HOCl), respectively, and found that the concentration of HOCl-alb with the 1 : 140 modification matched that in the plasma of diabetic rats, which were 32.68 *μ*M and 43.45 *μ*M, respectively [18]. The biological effects of HOCl-alb with the 1 : 140 modification were also comparable to those observed in uremic patients at the same doses. Obvious apoptosis of podocytes was noted after 48 h of stimulation [20].* In vivo*, NOXs activation, and MCP-1 expression were increased by repeated injection of HOCl-alb in diabetic rats [18], and* in vitro*, HOCl-alb induced podocyte apoptosis and NF-*κ*B activation [17]. Therefore, HOCl-alb with the 1 : 140 modification was used for subsequent experiments.

We found that HOCl-alb upregulated ICAM-1 protein and mRNA expression in human vascular endothelial cells* in vitro*. Because native Alb had no such effect, these biological actions were caused by the HOCl modification rather than by Alb itself. ICAM-1 molecules contain an extracellular immunoglobulin-like domain and act as ligands for the LFA-1 and MAC-1 molecules expressed on the surfaces of leukocytes. This mediates the adhesion between leukocytes (especially monocytes) and vascular endothelial cells and thus leukocyte activation, triggering the inflammatory response. The vascular inflammatory reaction is characterized by chemical chemotaxis, leukocyte adhesion, and migration, causing endothelial dysfunction, blood-vessel remodeling, and vascular disease [6]. We also found that HOCl-alb upregulated ICAM-1 expression in HUVECs* via* a signaling cascade involving MAPKs and NF-*κ*B. MAPKs are an important signaling system that mediates many cellular reactions. The ERK1/2 pathway (RAS–RAF–MEK1/2–ERK1/2) mainly regulates cell proliferation and differentiation, participates in cellular reactions caused by stress, bacterial products, and inflammatory mediators, and is closely associated with the inflammatory response. The p38^MAPK^ pathway is usually activated under stress conditions, such as LPS, inflammatory factors (interleukin-1 [IL-1], tumor necrosis factor-*α* [TNF-*α*]), and cell stressors (ultraviolet radiation, extracellular hyperosmosis, and thermal shock), and is involved in the release of a variety of inflammatory cytokines (including IL-1, TNF-*α*, and IL-6). NF-*κ*B is a reactive-oxygen-sensitive transcription factor [21], which regulates the expression of various genes, especially those related to the defense system and the inflammatory response. Previous data have illustrated that HOCl-alb induced upregulation of proinflammatory mediator monocyte chemoattractant protein-1 (MCP-1)* via* activation of ERK1/2 in HEK 293 cell lines [16] and promoted MCP-1 expression* via* NF-*κ*B activation in podocytes [17], which were consistent with our data. Firstly, we demonstrated that HOCl-alb activates ERK1/2 and p38^MAPK^ (but has no effect on JNK) in a time-dependent manner, and specific inhibitors of ERK1/2 and p38^MAPK^ both inhibited the upregulation of ICAM-1 expression induced by HOCl-alb, suggesting that the upregulation of ICAM-1 expression may depend on ERK1/2 and p38^MAPK^. Secondly, we observed the effects of HOCl-alb on NF-*κ*B in HUVECs with immunofluorescence, western blotting, and EMSA. The findings that NF-*κ*B was activated by HOCl-alb and translocated from the cytoplasm to the nucleus and that HOCl-alb-induced-ICAM-1 expression was inhibited by SN50, an inhibitor of the nuclear migration of NF-*κ*B, indicate that the HOCl-alb-induced upregulation of ICAM-1 expression depends on NF-*κ*B. Thirdly, specific inhibitors of ERK1/2 and p38^MAPK^ inhibited NF-*κ*B activation, but a specific inhibitor for NF-*κ*B did not inhibit ERK1/2 and p38^MAPK^ activation, suggesting that the activation of NF-*κ*B requires ERK1/2 and p38^MAPK^ but that the activation of ERK1/2 and p38^MAPK^ does not require NF-*κ*B. Therefore, ERK1/2 and p38^MAPK^ are signals upstream from NF-*κ*B. Lastly, the phosphorylation of ERK1/2 induced by HOCl-alb was not blocked by the p38^MAPK^-specific inhibitor and the ERK1/2-specific inhibitor had no effect on p38^MAPK^ activity, suggesting that ERK1/2 and p38^MAPK^ act in two parallel signaling pathways.

NOXs activity increases reactive oxygen, which acts as a second messenger to regulate an intracellular signaling cascade. MAPKs and NF-*κ*B are both oxygen-sensitive signaling systems, so we assumed that apocynin, a NOXs inhibitor, would inhibit ERK1/2 and the NF-*κ*B pathways activated by HOCl-alb. Cells were preincubated with apocynin and then stimulated with HOCl-alb. ERK1/2 and NF-*κ*B activation were inhibited and ICAM-1 expression decreased significantly. These were the most important findings in the present study and were consistent with the fact that apocynin at 500 *μ*M prevented NOXs-mediated LPS-induced-ICAM-1 expression in human pulmonary endothelial cells [21] and HOCl-alb triggered intracellular superoxide by activation of NOXs and apoptosis in podocytes were ameliorated by apocynin [20]. Our previous data also demonstrated that HOCl-alb-treated animals displayed a significant increase in renal expression of MCP-1, NOXs-dependent superoxide generation, and cell membrane translocation of NOXs subunits p47^phox^ and gp91^phox^ and all these perturbations could be prevented by the NOXs inhibitor apocynin [18], indicating that NOXs were participants in HOCl-alb-aggravated biological effects. As apocynin prevented MAPK signaling in vascular cells* via* NOXs-independent pathways at the concentrations of >100 *μ*M [30], we observed the effects of DPI, another inhibitor of NOXs, on ICAM-1 induced by HOCl-alb and found that DPI inhibited HOCl-alb-induced MAPKs activation and ICAM-1 expression. NOXs activation produced superoxide. Our findings that apocynin, DPI (NOXs inhibitors), and SOD (a scavenger of superoxide) all prevented HOCl-alb-induced ROS and superoxide generation strengthened the important effects of NOXs-derived superoxide anion radicals on the signaling actions of HOCl-alb in endothelial cells. Moreover, SOD also inhibited the phosphorylation activity of both ERK1/2 and p38^MAPK^ and the upregulation of ICAM-1 induced by HOCl-alb. These results suggest that NOXs are involved in the HOCl-alb-induced upregulation of ICAM-1 expression in HUVECs and that the activation of ERK1/2, p38^MAPK^, and NF-*κ*B required NOXs. In addition, the inhibition of HOCl-alb-induced ICAM-1 and the signaling cascade by apocynin is attributed to the action on NOXs.

It is known that NADPH oxidase in phagocytic cells is composed of two membrane-bound elements gp91^phox^ (also known as NOX_2_) and p22^phox^, three cytosolic proteins p40^phox^, p47^phox^, and p67^phox^, and Rac a small G-protein. The activation requires phosphorylation of cytosolic subunit p47^phox^, translocation to the cell membrane, and assembly with membrane-bound subunits. Seven isoforms of NOX have been identified in mammals: NOX_1_, NOX_2_ (formerly gp91^phox^), NOX_3_, NOX_4_, NOX_5_, DUOX_1_, and DUOX_2_. Four of these NOXs—NOX_1_, NOX_2_, NOX_4_, and NOX_5_—are expressed in endothelial cells. Similar to that in phagocytic cells, endothelial NOX_1_ and NOX_2_ require p22^phox^, p47^phox^, and p67^phox^ for their activation. NOX_4_ generates mainly H_2_O_2_. It also requires p22^phox^ but apparently does not associate with Rac1 or any other cytosolic subunits needed for activation of NOX_1_ and NOX_2_. NOX_5_ appears to produce superoxide in the absence of other “phox” or Rac subunits. It contains an N-terminal calmodulin-like domain with four binding sites for Ca^2+^; thus, the activation of NOX_5_ can be directly modulated by changes in intracellular [Ca^2+^] [15, 31]. However, it is unclear which isoform is involved in NOXs activation induced by HOCl-alb. As apocynin inhibits NOXs by preventing p47^phox^ translocation thereby inhibiting the assembly of functional NOXs [31, 32], does this mean that NOX_1_ and NOX_2_ but not NOX_4_ and NOX_5_ are involved? While this is out of the scope of the current study, further studies are required to identify the actual isoforms responsible for the NOXs-mediated proinflammatory signaling cascade in vascular endothelial cells exposed to HOCl-Alb.

## 5. Conclusion

HOCl-alb upregulates ICAM-1 protein and mRNA expression in vascular endothelial cells in a time- and dose-dependent manner. The pathobiological effects of HOCl-alb on endothelial cells are caused by the activation of a redox-sensitive intracellular signaling cascade involving ERK1/2 and p38^MAPK^, and culminating in NF-*κ*B. The upstream part of this pathway involves NOXs activation. As a specific inhibitor of NOXs, apocynin inhibits the pathological biological effects induced by HOCl-alb. NOXs are important interventional targets, and blocking NOXs-related pathways might protect against endothelial dysfunction and vascular diseases (Figure 8).

## Figures and Tables

**Figure 8:**